# Recent advances in heart transplantation

**DOI:** 10.12688/f1000research.14737.1

**Published:** 2018-07-05

**Authors:** Michelle M Kittleson

**Affiliations:** 1 Smidt Heart Institute at Cedars-Sinai, Los Angeles, California, USA

**Keywords:** Heart transplantation, organ allocation, transplant rejection, sensitization, crossmatch

## Abstract

Despite advances in medical and electrical therapies for heart failure, morbidity and mortality remain high and patients often progress to end-stage heart failure. Over the last five decades, heart transplantation is considered a standard therapy for select patients with end-stage heart failure. However, while heart transplantation has become a treatment of choice for end-stage heart failure, challenges still exist for improvement in the short- and long-term outcomes. While there is an increase in the number of patients with end-stage heart failure, the number of donor organs remains a major limiting factor. Heart transplantation candidates in the current era are also more complex: older, antigen-sensitized, and on mechanical circulatory support at the time of listing and transplant. Such potential heart transplant recipients have an increased chance of problems, including antibody-mediated rejection and primary graft dysfunction. Recent advances could address the current challenges and include: 1) attempts to expand the pool of donor hearts; 2) changes in heart transplantation allocation policy allowing for more equitable organ distribution; and 3) advances in the management of antibody sensitization. Developments in these areas could result in improved survival and quality of life for heart transplantation recipients.

## Introduction

Despite advances in medical and electrical therapies for heart failure, morbidity and mortality remain high and patients often progress to end-stage heart failure. Over the last five decades, heart transplantation is considered a standard therapy for select patients with end-stage heart failure. In the present era, one-year survival after heart transplantation is almost 90% and has a conditional half-life of 13 years ^1^, which is superior to that of end-stage heart failure.

The number of patients with heart failure requiring advanced therapies is growing, while the number of donor organs remains a constant and limiting factor ^2^. Heart transplant (HTx) candidates of the current era are also more complex. Increasing numbers are aged 65 years or more ^3^, have mechanical circulatory support ^3^, and have higher levels of antibodies to human leukocyte antigens (HLA), i.e. “sensitization” ^4^. Due to all these issues, these HTx candidates of the modern era are at increased risk for poor outcomes, including primary graft dysfunction and antibody-mediated rejection ^1, 2, 5^. The latest developments might be able to counter existing problems: 1) attempts to expand the pool of potential organ donors; 2) changes in the heart transplant donor allocation policy to allow for more equitable organ distribution; and 3) management of sensitized HTx candidates. Such advances could produce an increase in and a fairer allocation of donor organs and improved quality of life and survival for HTx recipients.

## Expanding the donor pool

### Extended criteria donors

Currently, fewer than 50% of potential donors in the United States become actual organ donors ^6^. Initiatives have been taken to increase the utilization rate ^7^. Using less-than pristine donor hearts, so-called extended criteria donors, is one option to expand the donor pool. These hearts may be used for higher-risk recipients, such as those who are older, over age 65, with diabetes, renal dysfunction, or peripheral vascular disease. Considerable evidence shows that extended criteria donor hearts that may result in favorable post-HTx survival continue to be underutilized. In a retrospective review in California from 2001 to 2008 looking at 1872 possible organ donors found predictors of non-use to be age >50 years, female gender, fatal cerebrovascular accident, hypertension, diabetes mellitus, an elevated troponin, left ventricular dysfunction (ejection fraction <50%), left ventricular hypertrophy, and regional wall motion abnormalities. However, when such so-called extended criteria donor hearts are used for transplantation, outcomes are generally comparable ^8– 11^.

### Limiting cold ischemic time

The donor pool could be expanded by limiting cold ischemic time. An *ex vivo* perfusion platform would allow the donor heart to be kept in a warm, beating state while being transported until the time of implantation. Small registry studies have demonstrated its safety ^12^. In a randomized trial examining the safety of an *ex vivo* platform, donor hearts managed with either the Organ Care System or standard cold storage were randomized to 130 patients. There was no difference in 30-day patient survival rates, 30-day graft survival rates, or serious adverse events ^13^.

The *ex vivo* perfusion platform may be particularly beneficial when used in conjunction with extended criteria donor hearts. Such donors, including those of older age, with left ventricular hypertrophy or moderately reduced ejection fraction, are more susceptible to injury with long ischemic time. Further study is needed to demonstrate the specific benefit of an *ex-vivo* perfusion platform in this population.

## Heart transplant allocation policy

### The impetus for a new approach

The allocation of donor hearts in the United States appears to be fair, as those patients who are sickest and who have been waiting the longest are the first to be considered in the event of a donor heart becoming available ( Table 1). However, changes in the HTx landscape have motivated efforts to improve the current system ^14, 15^: there is an imbalance between candidates awaiting transplantation and available donors, the sickest patients have unacceptably high mortality, and advances in mechanical circulatory support have decreased mortality in these transplant candidates.

**Table 1.** Current status codes for heart transplant allocation *.

Status Code	Criteria
Status 1A	● ECMO ● IABP ● Inpatient Total Artificial Heart (TAH) ● Mechanical ventilation ● Continuous infusion of a single high-dose intravenous inotrope or multiple intravenous inotropes, and with continuous hemodynamic monitoring of left ventricular filling pressures ● LVAD, RVAD, or BiVAD for 30 days ● Mechanical circulatory support with significant device-related complications (thromboembolism, device infection, mechanical failure, or life-threatening ventricular arrhythmias).
Status 1B	● Uncomplicated LVAD, RVAD, BiVAD after 30 days have been used ● Outpatient TAH ● Continuous infusion of intravenous inotropes
Status 2	● Candidates not meeting 1A or 1B criteria
Status 7	● Temporarily inactive, most often due to infection

BiVAD, biventricular assist device; ECMO, extracorporeal membrane oxygenation; IABP, intra-aortic balloon pump; LVAD, left ventricular assist device; RVAD, right ventricular assist device; TAH, total artificial heart

*Data from Organ Procurement and Transplantation Network Policy 6.1.

These alterations have uncovered two significant difficulties with the present status criteria. First, the system offers inadequate resolution. Status 1A includes the following groups with equal urgency: patients who receive extracorporeal membrane oxygenation (ECMO) and continuous intravenous inotropic support and hemodynamic monitoring. However, potential HTx recipients on ECMO support have increased mortality compared with candidates on inotropic support with continuous hemodynamic monitoring. Thus, these two groups of patients should not have the same priority, though they do under the current three-tiered system. The current system also makes no allowance for tenuous transplant candidates who do not qualify for Status 1A listing, including candidates with complex congenital heart disease, restrictive or infiltrative cardiomyopathies, or refractory ventricular tachycardia ^14, 15^.

### Making changes to the heart allocation policy

There are seven statuses in the new allocation policy ( Table 2) ^16^. Proposed statuses 1–3 are generally defined by current Status 1A criteria, proposed Status 4 is generally defined by current Status 1B, and proposed Status 5 and 6 are covered by current Status 2 criteria. The most significant alteration is the classification of patients within the current 1A status into three groups of decreasing acuity. In addition, the suggested policy tackles potentially underserved groups, such as adults with congenital heart disease, restrictive/hypertrophic cardiomyopathy, and re-transplantation in a separate tier above current Status 2 patients.

**Table 2.** Proposed new tiers for heart allocation model.

Proposed new tiers	Corresponding current tiers
1 i. VA ECMO	Status 1A
ii. Non-dischargeable BiVAD	Status 1A or 1B
iii. MCS with life-threatening ventricular arrhythmia	Status 1A
2 i. Non-dischargeable LVAD	Status 1A
ii. TAH, BiVAD, or RVAD	Status 1A or 1B
iii. MCS with device malfunction	Status 1A
iv. Percutaneous endovascular MCS device	Status 1A
v. IABP	Status 1A
vi. VT or VF	Status 1A
3 i. Dischargeable LVAD for discretionary 30 days	Status 1A
ii. Multiple inotropes or single inotrope with continuous hemodynamic monitoring	Status 1A
iii. MCS with hemolysis	Status 1A
iv. MCS with pump thrombosis	Status 1A
v. MCS with right heart failure	Status 1A
vi. MCS with device infection	Status 1A
vii. MCS with mucosal bleeding	Status 1A
viii. MCS with aortic insufficiency	Status 1A
ix-xi. VA ECMO, percutaneous endovascular circulatory support devices, or IABP after 14 days	Status 1A
4 i. Dischargeable LVAD without discretionary 30 days	Status 1B
ii. Inotropes without hemodynamic monitoring	Status 1B
iii. Congenital heart disease	NA
iv. Ischemic heart disease with intractable angina	NA
v. Amyloidosis, hypertrophic, or restrictive cardiomyopathy	NA
vi. Retransplant	NA
5 Combined organ transplants	NA
6 All remaining candidates	Status 2
7 Inactive/not transplantable candidates	Status 7/inactive

BiVAD, biventricular assist device; ECMO, extracorporeal membrane oxygenation; IABP, intra-aortic balloon pump; LVAD, left ventricular assist device; MCS, mechanical circulator support; RVAD, right ventricular assist device; TAH, total artificial heart; VA, venoarterial.

This table was adapted with permission from Meyer DM, Rogers JG, Edwards LB, *et al*., The future direction of the adult heart allocation system in the United States. Am J Transplant 2015;15:44-54 and https://optn.transplant.hrsa.gov/media/2028/thoracic_policynotice_201612.pdf (accessed May 24, 2017).

**Figure 1.**
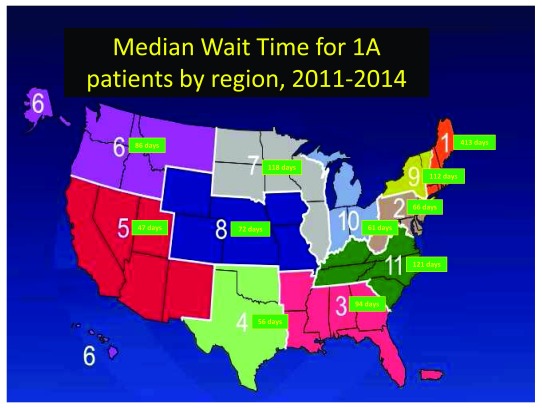
Regional wait time variation. There is significant variation by region, as defined by the United Network for Organ Sharing (UNOS), in the median wait time for status 1A patients. Data from https://optn.transplant.hrsa.gov/data/view-data-reports/regional-data/.

The novel allocation system deals with geographical inequality in organ distribution ( Figure 1) by broader sharing for the highest tier patients. The two highest-acuity groups will draw organs from a 500-mile radius in the first round of organ allocation instead of using a stepped approach.

This system was approved by the United Network of Organ Sharing Board and Organ Procurement and Transplant Network in December 2016. It will go into effect in the last quarter of 2018. No system can be perfect, but these efforts allow for more equitable distribution of this scarce resource and achieve the primary goal, whereby the most critically ill patients can receive transplantation before their window of viability closes.

## Advances in the diagnosis of rejection

### Allomap: a tool utilizing peripheral blood gene expression

Despite the fact that endomyocardial biopsy is usually a simple procedure, the morbidity associated has motivated development of other means to diagnose rejection. The gene expression profile test (AlloMap®, CareDx Inc, San Francisco, CA), an 11-gene expression signature from peripheral blood mononuclear cells, has a high negative predictive value for cellular rejection ^17^ and is noninvasive. In randomized trials, gene expression profile was non-inferior to an endomyocardial biopsy in the diagnosis of cellular rejection ^18^ and also useful early post-transplant ^19^. One role of the gene expression profile is to screen patients at low risk for cellular rejection at pre-determined intervals with biopsies performed only in cases in which the gene expression profile score is abnormal. However, patients with risk factors for antibody-mediated rejection cannot be tested with gene expression profile screening as the technique can only be used to assess cellular rejection.

### Cell-free DNA

One more novel technique that can be used for noninvasive diagnosis of rejection uses cell-free DNA. Cell-free donor-derived DNA can be detected in the blood and urine of organ transplant recipients ^20, 21^ This cell-free DNA may be a potential marker for noninvasive diagnosis of graft injury, as cellular and antibody-mediated rejection events are associated with increased levels of cell-free donor-derived DNA ^22^.

### Biopsy-derived endomyocardial gene expression

As mentioned above, the Allomap relies on peripheral blood gene expression profiling to refine diagnostic accuracy and does not detect antibody-mediated rejection (AMR). A recent study, however, studied mRNA extracted from endomyocardial biopsy samples and hybridized to a microarray system. Differences in AMR-selective gene expression classified AMR were linked with disease activity and ISHLT AMR grade ^23^. This advance has the potential to refine diagnostic accuracy and may in the future provide insight into the management of AMR ^24^.

## Sensitization

### Identification and quantification of anti-HLA antibodies

**Figure 2.**
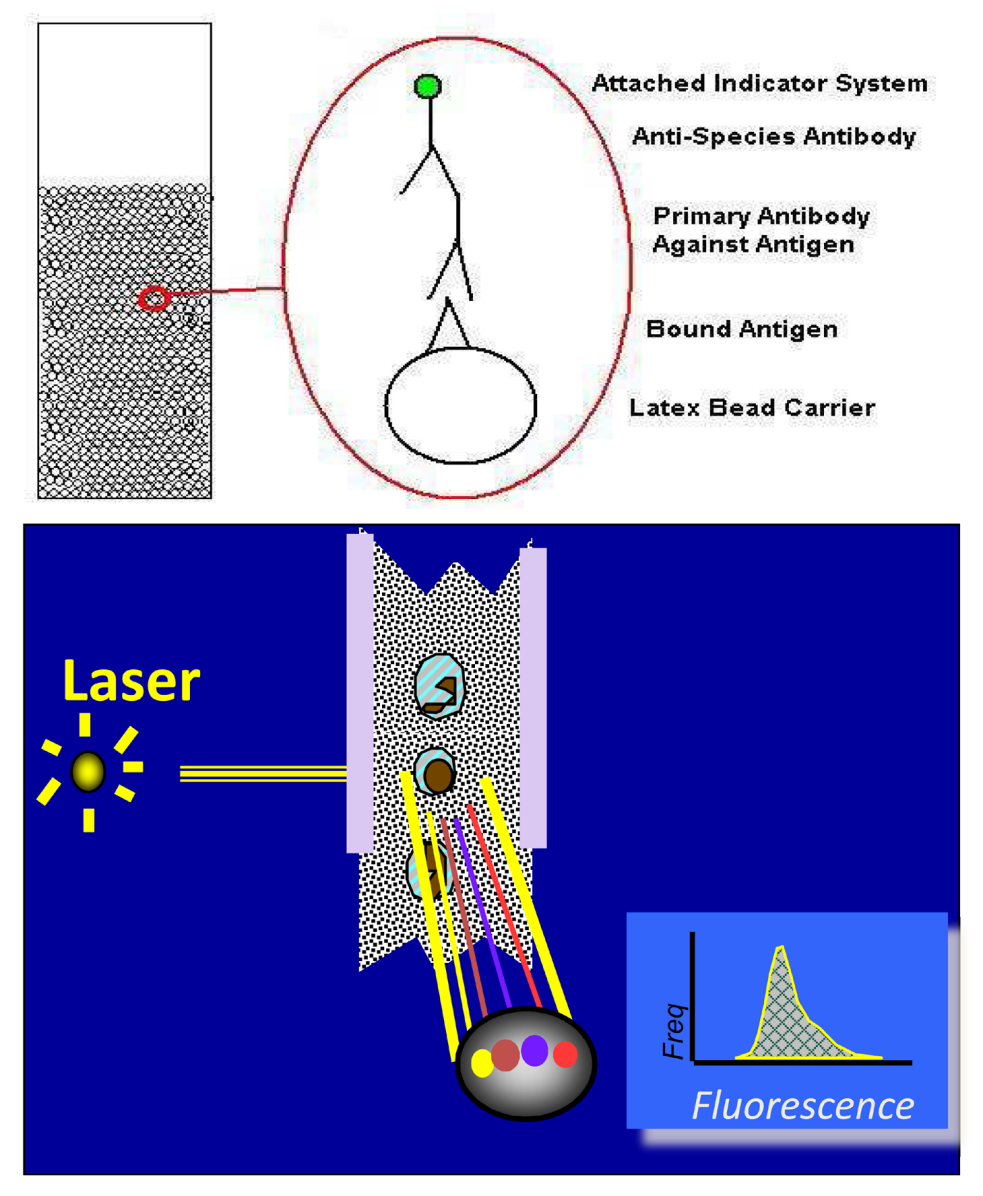
The detection of anti-HLA antibodies utilizing solid-phase assays. Antibodies bind fluorescent-tagged antigens. A flow cytometer identifies anti-HLA antibodies and provides information on antibody strength and potential cytotoxicity.

The detection and measurement of anti-HLA antibodies is accomplished using solid phase assays ( Figure 2). Quantification is clinically relevant, as antibodies of greater intensity *in vitro* are potentially more cytotoxic *in vivo*. The presence of high-level anti-HLA antibodies (usually median fluorescent intensity [MFI] above 3,000–5,000) are considered potentially cytotoxic ^24^.

However, not all high-intensity antibodies are detrimental to graft function and the fact that donor-specific antibodies have the ability to fix complement might be a superior way of signifying cytotoxicity ^25, 26^. The classical complement pathway is activated first by C1q binding to antibodies, as C1q is the first component of the pathway. After C1q activation, the complement cascade results in formation of the membrane attack complex and ultimately leads to cell lysis and death. In fact, antibodies with the ability to bind C1q are more likely to be cytotoxic ^25, 27^. The C1q assay is not currently available in all centers, so considering only antibodies that are strong binding by MFI after a 1:8 or 1:16 dilution may offer comparable information ^26^.

**Figure 3.**
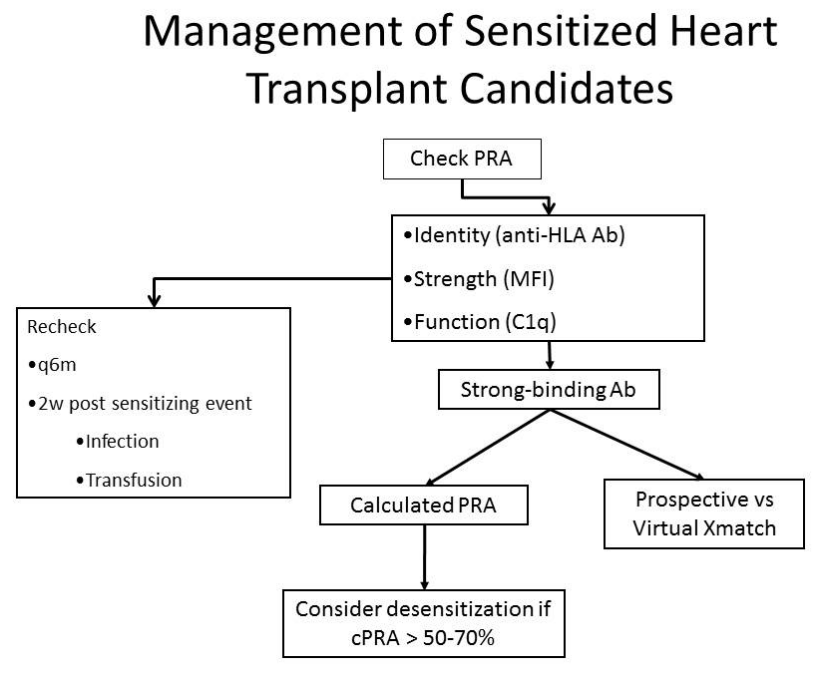
Management of the sensitized patient. Sensitized patients are those with a positive panel reactive antibody (PRA) screen; we consider a PRA > 10% to be positive. The next step is to determine the identity and intensity of the anti-HLA antibodies. The results are used to determine the calculated PRA and the need for a virtual crossmatch. If the calculated PRA is above 50–70%, desensitization therapy may be used. This figure was reprinted with permission from 28.

Figure 3 illustrates one approach to the identification and quantification of antibodies against HLA and how management decisions are made in sensitized heart transplant candidates.

### Approach to the crossmatch

Prior to transplantation, the detection of anti-HLA antibodies is important to avoid hyperacute rejection: one would avoid donors with HLA correlating with high-level anti-HLA antibodies in the prospective recipient. One way to prevent hyperacute rejection is a prospective crossmatch, in which the potential recipient’s serum and donor cells are mixed to evaluate for complement-dependent cytotoxicity. However, this restricts the donor pool based on the location of the candidate’s serum, and so decreasing the number of possible donors.

Thus, the virtual crossmatch has essentially replaced the prospective crossmatch. HLA correlating with the candidate’s high-level anti-HLA antibodies are recorded as “avoids” in the United Network of Organ Sharing (UNOS) database. In this manner, potential donors with such HLA are not considered. This method has proven feasible in HTx ^29^.

### The calculated PRA

The identity and the intensity score (given by Luminex single-antigen bead assay) of anti-HLA antibodies is also valuable information for deciding which potential heart transplant recipients require desensitization, as expressed as the calculated PRA (cPRA) ^24, 30^. The cPRA is the frequency of HLA defined as unacceptable in the donor population. For example, a HTx candidate with multiple anti-HLA antibodies might have a cPRA of 90%. This would mean that of all potential donors only 10% would be a match. cPRA highlights the fact that some high-level anti-HLA antibodies, the more common ones, will impact the ability to identify a suitable donor heart more than less common anti-HLA antibodies ^31^. If the cPRA is above 50–70%, therapies to reduce antibody levels may be administered.

### Approach to desensitization

**Figure 4.**
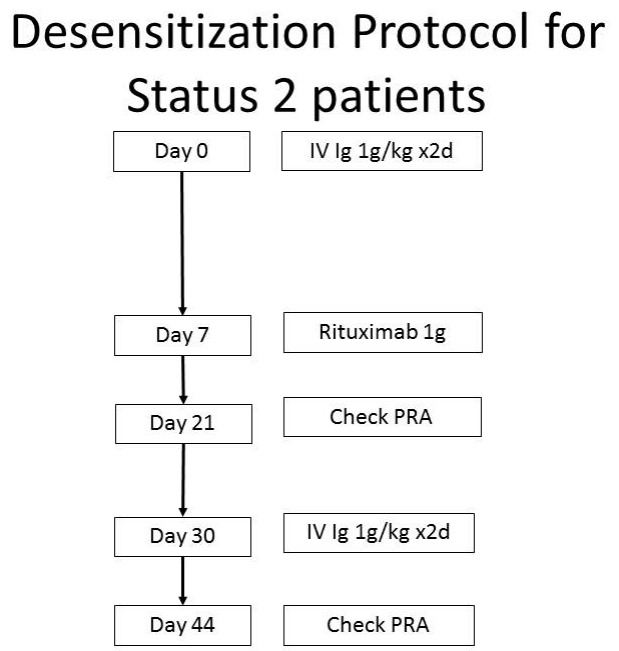
Desensitization protocol. The treatment of circulating antibodies depends on the cPRA. Treatment is considered for those patients with cPRA >50–70%. This figure was modified from 32.

Management of the sensitized patient includes protocols that target antibodies by inactivation (intravenous immune globulin [IV Ig] ^32^), removal (plasmapheresis), and reduced production (rituximab ^32^) and bortezomib ^33^). At our center, the desensitization process usually starts with a modified protocol built around one established for desensitization of kidney transplant recipients ( Figure 4) ^32^.

**Figure 5.**
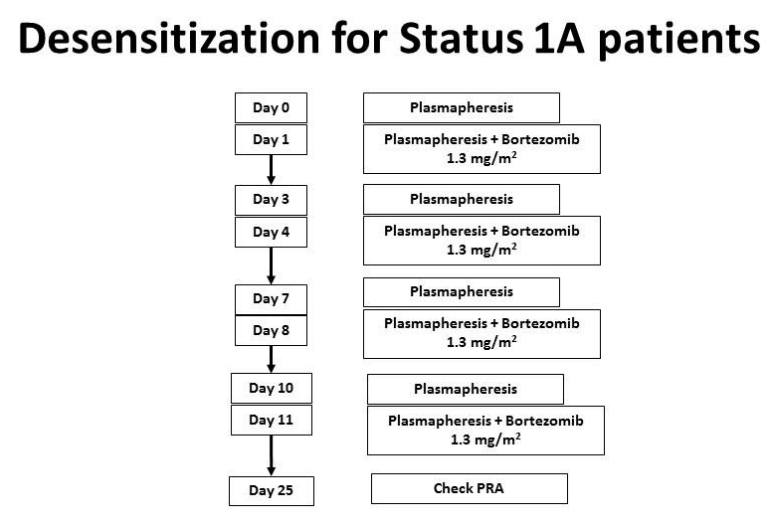
Desensitization of Status 1A patients or those with refractory antibodies. Bortezomib is used for Status 1A patients or those with antibodies that do not respond to IV Ig and rituximab. This regimen will lower antibodies more effectively. This figure was reprinted with permission from Kittleson MM, Kobashigawa JA. Management of the Highly Sensitized Patient Awaiting Heart Transplant. January 8, 2015. Available at: http://www.acc.org/latest-in-cardiology/articles/2014/12/22/17/07/management-of-the-highly-sensitized-patient-awaiting-heart-transplant-expert-analysis (accessed April 9, 2018).

If the combination of intravenous immune globulin and rituximab is ineffective in decreasing the cPRA below 50%, or if swift desensitization is required (i.e. a patient is listed as status 1A), then one may use bortezomib, a proteasome inhibitor targeting plasma cells ^33^. To increase effectiveness, bortezomib can be combined with plasmapheresis ( Figure 5).

***Eculizumab.*** Pre-transplant interventions with IV Ig, rituximab, and bortezomib can reduce antibody levels so that it is possible to find an acceptable donor. However, for some patients such measures are not effective. Hyperacute rejection can still occur at the time of transplantation due to cytotoxic anti-HLA antibodies. This can happen if donor-specific antibodies were mistakenly classified as not cytotoxic by virtual crossmatch or if such antibodies were not present at the time banked blood was stored for a prospective crossmatch. Plasmapheresis may be used in the operating room at the time of transplantation in this setting. We have also found that eculizumab offers further insurance and protection against hyperacute rejection.

The monoclonal antibody eculizumab blocks the last component of the complement cascade. This cascade is triggered by antigen-antibody complexes and results in formation of the membrane attack complex and ultimately cell death. Eculizumab specifically binds to the terminal complement component 5 (C5), and thus eventually blocks the production of the terminal complement complex C5b-9. Eculizumab is FDA-approved for use in two complement-mediated conditions, paroxysmal nocturnal hemoglobunuria and atypical hemolytic-uremic syndrome. However, it also has benefit in sensitized kidney transplant recipients ^34^.

## Conclusion

The boundaries of HTx continue to expand to higher-risk candidates—older, on mechanical circulatory support, and with HLA antibody sensitization—and our goal remains to maintain favorable outcomes utilizing this scarce resource. The advances outlined here, from efforts to expand the donor pool, revision of the HTx allocation policy, newer ways to diagnose rejection, to developments in the detection and care of sensitized HTx candidates, will bring about improvements in survival and quality of life of end-stage patients who have HTx.

## Abbreviations

AMR, antibody-mediated rejection; cPRA, calculated panel reactive antibodies; ECMO, extracorporeal membrane oxygenation; HTx, heart transplantation; IV Ig, intravenous immune globulin; MFI, median fluorescent intensity; PRA, panel reactive antibodies.
